# High-throughput profiling identifies clinically actionable mutations in salivary duct carcinoma

**DOI:** 10.1186/s12967-014-0299-6

**Published:** 2014-10-25

**Authors:** Bo Mi Ku, Hyun Ae Jung, Jong-Mu Sun, Young Hyeh Ko, Han-Sin Jeong, Young-Ik Son, Chung-Hwan Baek, Keunchil Park, Myung-Ju Ahn

**Affiliations:** Samsung Biomedical Research Institute, Seoul, Korea; Division of Hematology-Oncology, Department of Medicine, Samsung Medical Center, Sungkyunkwan University School of Medicine, 50 Irwon-dong, Gangnam-gu, Seoul, 135-710 Korea; Department of Pathology, Samsung Medical Center, Sungkyunkwan University School of Medicine, Seoul, Korea; Department of Otorhinolaryngology-Head and Neck Surgery, Samsung Medical Center, Sungkyunkwan University School of Medicine, Seoul, Korea

**Keywords:** Salivary duct carcinoma, Next-generation sequencing, Molecular markers, PIK3CA, ERBB2, EGFR

## Abstract

**Background:**

Salivary duct carcinoma (SDC) is a highly aggressive subtype of salivary gland cancers and there is no established standard therapy for this disease. Thus, development of molecular markers for SDC will be important to guide the diagnosis and therapy of this aggressive tumor.

**Methods:**

We performed next-generation sequencing using the Ion Torrent AmpliSeq cancer panel, which explores the mutational status of hotspot regions in 50 cancer-associated genes, and we analyzed copy number variations (CNVs) of 21 genes by NanoString nCounter for 37 patients with SDC. Fluorescent in situ hybridization was also conducted to confirm *ERBB2* gene amplification. Clinical records and tumor histopathology of the patients were retrospectively reviewed.

**Results:**

Genetic alterations were detected in 29 of 37 (78.3%) tumors, including mutations in *PIK3CA* (*N* = 9, 24.3%), *ERBB2* (*N* = 4, 10.8%), and *EGFR* (*N* = 4, 10.8%). To our knowledge, this is the first time that *ERBB2* mutations have been reported in this tumor type. Both *PIK3CA* and *ERBB2* mutation status were associated with poor overall survival, but without statistical significance. *ERBB2* amplification was strong and common in SDC and almost all cases also exhibited *EGFR* and *ERBB3* amplifications.

**Conclusions:**

This study reports the largest and most comprehensive analysis of DNA aberrations in SDC. Our results show that *PIK3CA* and/or *ERBB2* alterations in the development of SDC might be a useful diagnostic tool and could serve as a potential therapeutic target.

## Background

Salivary gland cancers (SGC) are rare, accounting for fewer than 5% of all cancers of the head and neck [1]. SGC present numerous morphologic, biologic, and clinically diverse entities. Salivary duct carcinomas (SDC) are a rare and aggressive subtype of SGC [2,3]. It bears a pathologic resemblance to ductal carcinoma of the breast and shares hormonal and molecular marker alterations with breast cancer [4]. SDC mainly arises *de novo* in the parotid gland and most patients are older than 50 years. The recurrence rate is high and metastases occur in lymph nodes, lung, liver, and bone [5]. Despite surgical resection and postoperative radiotherapy, SDC has an extremely poor prognosis [2,4] and most patients with SDC die from progressive disease within 3 years [2,6].

To date, several chemotherapeutic agents have been tested but none have shown significant efficacy [7]. Although there are limited prior molecular studies of SDC, many of these studies have suggested that overexpression of epidermal growth factor receptor 2 (ERBB2*,* also known as HER2) is a common event in this tumor type. Expression of ERBB2 has been linked to early local disease recurrence, distant metastasis, and poor survival [2,8]. In spite of the high incidence of *ERBB2* gene amplification, as detected by fluorescence in situ hybridization (FISH), there has been no meaningful or reproducible response to *ERBB2*-targeted therapy [4,9]. Furthermore, given the rarity of the disease, comprehensive analyses of biomarkers are limited. Thus, identifying the molecular genetic alterations in SDC should provide new opportunities for the development of therapeutics.

Significant improvements in sequencing technology and computational methods have led to the development of next-generation sequencing (NGS) platforms. Several NGS platforms are available for sequencing either targeted genomic regions or whole genomes/exomes to analyze a variety of disease-associated changes such as point mutations, insertions, deletions, and copy number variations (CNVs) [10]. These broad NGS applications have facilitated the discovery of novel genetic aberrations in disease and NGS may be a useful tool for mutation screening for diagnosis and discovery [11].

In the present study, we used an AmpliSeq cancer panel and NanoString nCounter assay to determine cancer-associated mutations and CNVs in 37 SDC patients. CNVs of *ERBB2* that were detected using NanoString nCounter assay were confirmed using FISH.

## Methods

### Tumor specimen and DNA extraction

All procedures involving tumor specimen were reviewed and approved by the Institutional Review Board (IRB) of Samsung Medical Center (SMC). Patient records/information was anonymized and de-identified prior to analysis. We retrospectively identified 48 patients who were diagnosed with SDC at Samsung Medical Center from January 1997 to April 2010. A total of 37 cases of SDC had adequate tissue available for DNA isolation. Genomic DNA was extracted from formalin-fixed paraffin-embedded tissue (FFPE) using the QIAamp DNA FFPE Tissue Kit (Qiagen). Purified DNA was quantitated using NanoDrop (Invitrogen Life Technologies) and Qubit (Invitrogen Life Technologies).

### Next-generation sequencing

Ion Torrent Ion AmpliSeq Cancer Hotspot Panel v2 (Life Technologies) was used to sequence hotspot regions in 50 frequently mutated tumor suppressor genes and oncogenes. For multiplex PCR amplification, 10 ng of DNA (quantified by Qubit™ Fluorometer) was used and the custom Ion AmpliSeq panel was processed with Ion AmpliSeq Library kit 2.0 according to the manufacturer’s instructions. The resulting amplicons were treated with FuPa Reagent to partially digest the primers and phosphorylate the amplicons. The amplicons were then ligated to the Ion Xpress™ Barcode Adapters (1–96 Kit) and template preparation was performed with the Ion OneTouch™ System using Ion OneTouch™ 200 Template Kit v2 DL. Sequencing was performed on Ion 316 chips using the Ion PGM™ 200 Sequencing Kit according to the manufacturer’s instructions. The raw signal data were analyzed using Torrent Suite v.4.0.2 (Life Technologies). The pipeline includes signaling processing, base calling, quality score assignment, adapter trimming, read alignment (Torrent Mapping Alignment Pregram; TMAP) to human genome (HG) 19 reference, mapping quality control, and coverage analysis. Variant calling was performed using the Torrent variant Caller 4.0 software. COSMIC DB, dnsnp137, and annovar were used as annotation program.

### Copy number alteration analysis

For detection of CNVs in salivary ductal carcinoma, a panel of customized gene probes was designed using NanoString nCounter technology and subsequently analyzed on the NanoString nCounter platform. NanoString probes were composed of the following 21 genes (in alphabetical order): *AURKA*, *CCND1*, *CCNE1*, *CDK4*, *CDK6*, *CDKN1A*, *CDKN2A*, *EGFR*, *ERBB2*, *ERBB3*, *FGFR1*, *FGFR2*, *IGF1R*, *KLF5*, *KRAS*, *MDM2*, *MET*, *MITF*, *MYC*, *PIK3CA*, *TNIK*. For the nCounter assay, 600 ng of genomic DNA was hybridized with the custom designed code set for 18 hours at 65°C and processed according to the manufacturer’s instruction. The data were normalized to the invariant control probes and to positive and negative controls in each hybridization reaction.

### Fluorescence in situ hybridization

To evaluate copy number changes in the *ERBB2/HER2* gene, a 4-μm section of FFPE tumor tissue was analyzed by dual-color FISH using the PathVysion HER2 DNA Prove Kit (Abbott Molecular), which includes HER2 Spectrum Orange and centromere 17 (CEP17) Spectrum Green.

### Statistical analysis

Patient characteristics were compared using chi-square and Fisher’s exact tests (categorical variables). Survival time was estimated using the Kaplan-Meier method and compared for statistical differences by log-rank analysis. Multivariate analysis was performed using stepwise cox proportional hazards regression modeling to assess the independent prognostic role of each clinicopathologic variable. Statistical analyses were performed using SPSS19.0 (SPSS Inc.) and statistical significance was considered to be *p* <0.05.

## Results

### Clinical characteristics of the cohort

Characteristics of all 48 patients are listed in Table 1. The median age was 59.3 years (range 39.3-81.6 years) and male patients were predominant (75%; 36/48). The primary tumor site was the parotid gland in 38 (80%) cases and the submandibular gland in 10 cases (20%). Regarding tumor stage, the majority (44%) of patients presented with stage IV disease. Surgery was performed for 41 patients (85.4%) and postoperative radiotherapy and/or chemotherapy were performed for 24 (50%) and 14 (29%) cases, respectively. Among patients who did not undergo surgery, 1 case was treated with concurrent chemo-radiotherapy, 2 cases with palliative chemotherapy, and 4 cases with palliative radiotherapy. Among 42 patients who underwent surgery, 19 cases (45.2%) had recurrence of disease at either locoregional or distant sites and 23 cases (54.8%) showed no evidence of disease. With a median follow-up of 44.7 months (range: 3.7-214.0 months), the median recurrence-free survival (RFS) time was 17.3 months (95% CI, 14.2-20.4) and median overall survival (OS) time was 76.2 months (95% CI, 15.7-136.7). Among the 48 patients, only 37 cases of SDC had adequate tissue available for DNA isolation.

**Table 1:** **Baseline characteristics**

**Total (N =48)**		**Patient (N)**
**Age** (Median/Range)		59.3 (39.3-81.6) years
**Sex** (Male : Female)		36 : 12
**Location**		
Parotid gland		38
Submandibular gland		10
**Stage**		
I		4
II		17
III		6
IV		21
**Treatment**		
Operation		3
Operation + RT		25
	Radiation dose (Median/Range)	5400 cGy (4000-6300 cGy)
Operation + CCRT		14
	Radiation dose (Median/Range)	5940 cGy (5400-6000 cGy)
	Chemotherapy regimen	
	Cisplatin	1
	DP	5
	FP	2
	CAP	4
	Unknown	2
CCRT		1 (Genexol/cisplatin)
Palliative chemotherapy		2 (CAP, XP)
Palliative radiotherapy		4
**Treatment response**		
No evidence disease		23
Stable disease		1
Recur or Progression		19/5
**Recurrence sites**		Total (N =19)
Locoregional recurrence		8
Lung		6
Bone		4
Liver		2
**RFS (Median)**		29.1 months
**OS (Median)**		76.2 months

RT: Radiotherapy; CCRT: Concurrent chemo-radiotherapy; DP: Docetaxel/Cisplatin; FP: 5FU/Cisplatin; CAP: Cyclophosphamide/Doxorubicin/Cisplatin; RFS: Recurrence-free survival; OS: Overall survival.

### Molecular profiling of SDC

**Figure 1:**
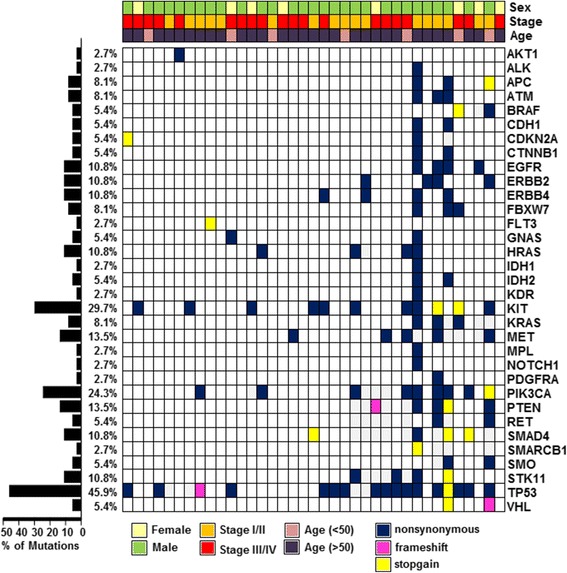
**Heatmap of the mutations found in 37 SDC samples.** In the upper panel, the first row indicates sex, the second row cancer stage, and the third row age. (Left) the histogram shows the percentage of mutations in each gene. (Right) The horizontal axis presents the complete dataset of patients and the vertical axis illustrates mutated genes.

To evaluate genetic abnormalities occurring in SDC, we applied NGS technology using the Ion Torrent Ion AmpliSeq Cancer Hotspot Panel. Among the detected mutations, only mutations annotated in the Catalogue of Somatic Mutations in Cancer (COSMIC) database were considered. Twenty-nine of 37 (78.3%) patients possessed more than one mutation in this analysis (Figure 1). Genes with identified mutations included *APC* (8 mutations), *ATM* (3), *EGFR* (7), *ERBB2* (4), *ERBB4* (5), *FBXW7* (3), *HRAS* (2), *KIT* (7), *KRAS* (4), *MET* (1), *PIK3CA* (15), *PTEN* (10), *SMAD4* (10), *STK11* (4), and *TP53* (33) (Table 2). With the exception of *TP53* and *KIT* mutations, the most frequently identified mutations were in *PIK3CA* (15 mutations in 9 patients). Three hotspot mutations (E542K, E545K, and H1047R) of *PIK3CA* were observed in 13.5% of patients (5/37). Although seven *EGFR* mutations were detected, there were no cases with activating *EGFR* mutations (exon 19 deletion or L858R), which are well-known gene abnormalities in non-small cell lung cancer. Interestingly, in addition to *ERBB2* amplification, which is known to be a common event in SDC [4], our results also revealed *ERBB2* mutations in four patients at four different loci: I767M, D769Y, G776V, and V842I. The *KRAS* mutations identified in this study were not in codons G12, G13, or Q61, which impair the intrinsic GTPase activity of RAS and sustain the activation of RAS signaling [12].

**Table 2:** **Mutations found in 37 SDCs by AmpliSeq**

**Gene**	**Accession**	**Number of mutations**	**Mutations**
APC	NM_000038	8	G1106R, A1347T, P1361L, E1374K, Q1378X, S1434N, G1466E, H1490Y
ATM	NM_000051	3	E848K, D1693N, A1742V
EGFR	NM_005228	7	V765M, A859T, A864V, E865K, E866K, A871T, G874S
ERBB2	NM_004448	4	I767M, D769Y, G776V, V842I
ERBB4	NM_005235	5	A287T, S341L, D609N, R612Q, P619L
FBXW7	NM_033632	3	G391D, D440N, R441W
HRAS	NM_176795	2	G13S, Q61R
KIT	NM_000222	7	D496N, M541L, W557X, E561K, W582X, A636V, A637F
KRAS	NM_004985	4	G15S, T35I, E49K, E63K
MET	NM_000245	1	N375S
PIK3CA	NM_006218	15	E65K, R88X, R108H, E110K, S326F, V344M, D350N, E542K, E545K, D1018N, A1020T, E1037K, M1043I, H1047R, G1049D
PTEN	NM_000314	10	W111X, D115N, G132S, M134I, R233X, R234Q, Q245X, E256K, T319fs, P339L
SMAD4	NM_005359	10	Q116X, R135X, Q256X, P356S, P356L, R496H, R497H, C499Y, W509X, R531W
STK11	NM_000455	4	Q170X, G171S, P281L, F354L
TP53	NM_001126112	33	A78V, P80L, A86V, S95F, V97A, P98L, R110C, S127fs, P128fs, K132R, C135W, V157G, R181C, Q192X, H193Y, R213Q, S215R, P219S, P223S, G226D, S241F, V272G, R273C, A276D, D281N, R282W, E285K, E286G, E287K, R290H, G293R, G302E, E339K

**Figure 2:**
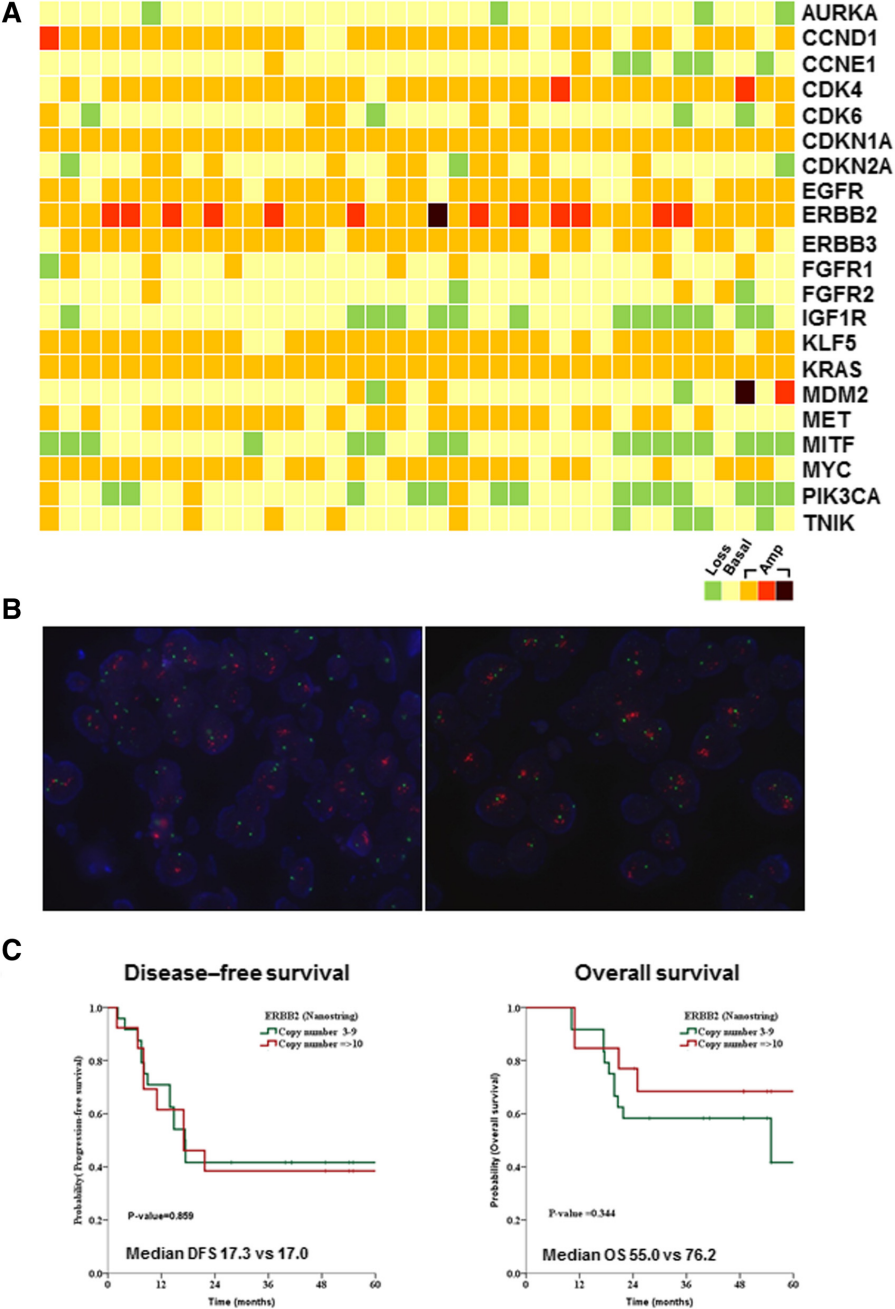
**Copy number variations in SDC. A**. Copy number variations of 37 SDC samples by the NanoSting nCounter Cancer Copy Number Variation Panel. The horizontal axis represents the complete dataset of patients and the vertical axis indicates examined genes. **B**. *ERBB2* gene amplifications were confirmed by FISH analysis, showing clusters of red signals (*ERBB2* probe) and 2 green probes (chromosome 17) per nucleus (DAPI staining). **C**. Disease-free survival (left) and overall survival (right) depicted according to the *ERBB2* gene amplification. Patients grouped by strong (copy number ≥10) and moderate (copy number <10) amplification of *ERBB2*.

The CNVs were also tested and validated by using NanoString nCounter technology. The results showed that 14 of the 21 genes had CNV, including both copy number amplifications and losses. These genes included *CCND1* (83.7%), *CDK4* (91.8%), *CDKN1A* (100%), *CDKN2A* (35.1%), *EGFR* (81%), *ERBB2* (100%), *ERBB3* (81%), *IGF1R* (37.8%), *KLF5* (86.4%), *KRAS* (100%), *MET* (64.8%), *MITF* (43.2%), *MYC* (72.9%), and *PIK3CA* (45.9%) (Table 3). Of note, *ERBB2* exhibited the highest prevalence of strong gene amplification in SDC (Figure 2A). Using FISH, we confirmed *ERBB2* gene amplification in 29 cases of SDC. Representative images of SDC cases exhibiting *ERBB2* gene amplification are illustrated in Figure 2B. As all patients in this cohort had *ERBB2* gene amplification (copy number >2), we classified these patients into moderate (copy number <10) and strong (copy number >10) amplification of *ERBB2*. When survival was analyzed according to amplification status of *ERBB2*, there was no difference in disease-free survival and overall survival between the two groups (Figure 2C).

**Table 3:** **The frequency CNV in 37 SDCs by NanoString nCounter analysis**

**Gene**	**Case no.**	**Copy number amplification no. (%)**	**Copy number loss no. (%)**	**Total CNV frequency no. (%)**
AURKA	37	0 (0)	4 (10.8)	4 (10.8)
CCND1	37	31 (83.7)	0 (0)	31 (83.7)
CCNE1	37	2 (5.4)	5 (13.5)	7 (18.9)
CDK4	37	34 (91.8)	0 (0)	34 (91.8)
CDK6	37	6 (16.2)	4 (10.8)	10 (27)
CDKN1A	37	37 (100)	0 (0)	37 (100)
CDKN2A	37	10 (27)	3 (8.1)	13 (35.1)
EGFR	37	30 (81)	0 (0)	30 (81)
ERBB2	37	37 (100)	0 (0))	37 (100)
ERBB3	37	30 (81)	0 (0)	30 (81)
FGFR1	37	8 (21.6)	1 (2.7)	9 (24.3)
FGFR2	37	3 (8.1)	2 (5.4)	5 (13.5)
IGF1R	37	0 (0)	14 (37.8)	14 (37.8)
KLF5	37	32 (86.4)	0 (0)	32 (86.4)
KRAS	37	37 (100)	0 (0)	37 (100)
MDM2	37	5 (13.5)	2 (5.4)	7 (18.9)
MET	37	24 (64.8)	0 (0)	24 (64.8)
MITF	37	0 (0)	16 (43.2)	16 (43.2)
MYC	37	27 (72.9)	0 (0)	27 (72.9)
PIK3CA	37	3 (8.1)	14 (37.8)	17 (45.9)
TNIK	37	5 (13.5)	4 (10.8)	9 (24.3)

Copy number amplification: copy number >2.

Copy number loss: copy number <2.

### Impact of molecular marker status on clinical outcomes

**Figure 3:**
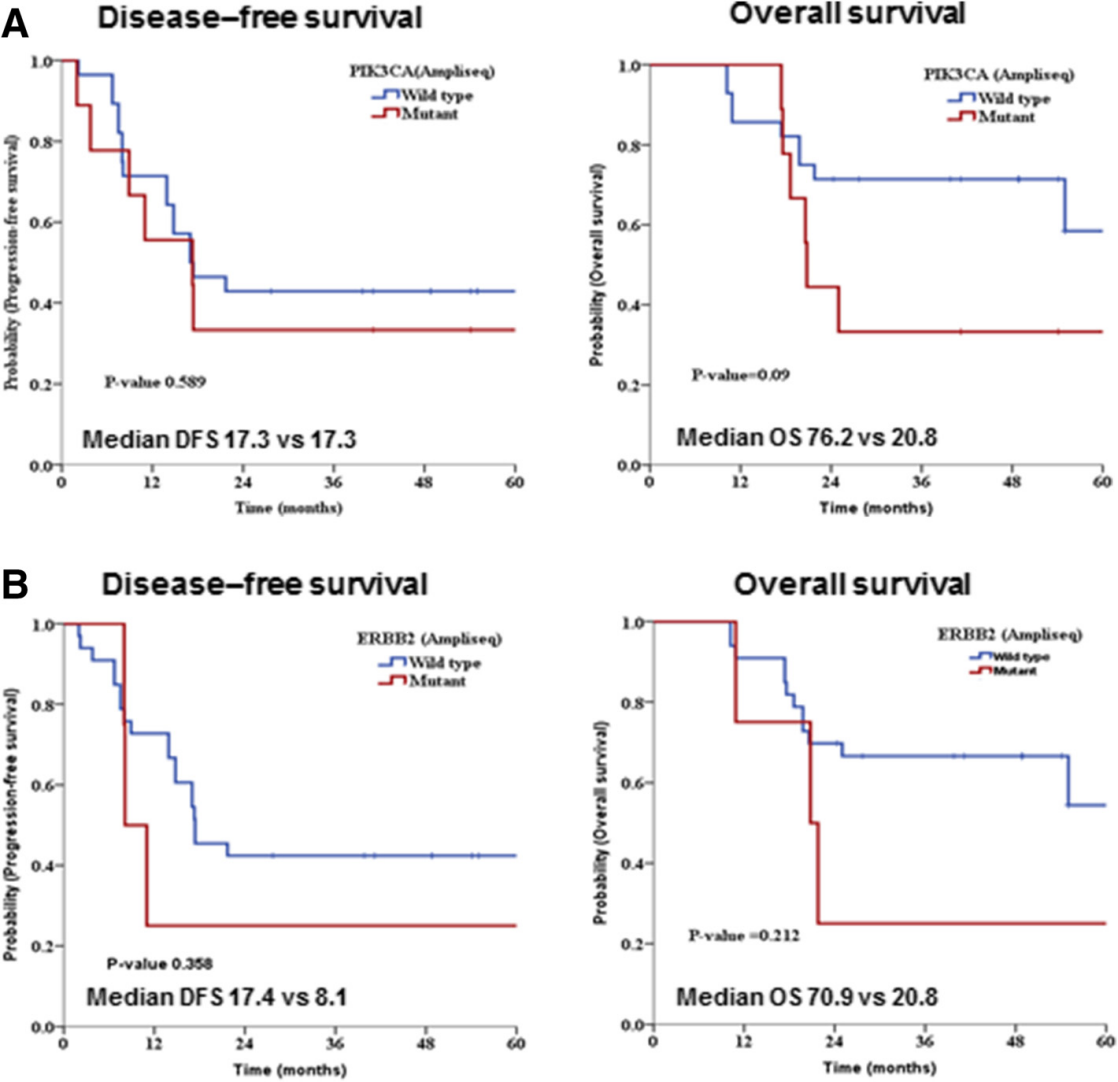
**Kaplan-Meier curves for disease-free survival (DFS) and overall survival (OS) analysis.** Observed survival of SDC patients is depicted according to the presence or absence of genetic abnormalities: **A**. mutations in *PIK3CA* and **B**. mutations in *ERBB2*. Statistical analysis revealed no significant differences but indicated a trend toward better survival for patients without genetic mutation.

Given the high incidence of *PIK3CA* or *ERBB2* gene abnormalities in this cohort, we investigated the association between molecular status and survival. Kaplan-Meier survival curves were plotted according to *PIK3CA* or *ERBB2* mutation status (Figure 3). In the case of *PIK3CA* (Figure 3A) or *ERBB2* (Figure 3B) mutation, both disease-free survival (DFS) and overall survival (OS) curves shifted down. Although there was a tendency toward lower survival in patients with gene mutation (median OS: 76.2 months for wild-type vs. 20.8 months for *PIK3CA* mutation; 70.9 months for wild-type vs. 20.8 months for *ERBB2* mutation), the association was not statistically significant.

## Discussion

Salivary duct carcinoma is an aggressive subtype of salivary gland cancers with histologic similarities to ductal breast carcinoma. Although patients can receive effective therapy including surgical resection followed by radiation therapy or chemotherapy, the high recurrence rate results in poor prognosis [4,7]. The most active single agents for SDC include cisplatin, cyclophosphamide, doxorubicin, and 5-FU. Response rates for combination chemotherapy are reported to be 15-50% [7]. Given the lack of promising therapeutics in SDC, in this study we aimed to investigate molecular features that can predict responses to therapy and characterize molecular markers for SDC by evaluating genetic status.

Consistent with previous studies, our patient population consisted mostly of males (75%) older than 50 years, who presented with advanced disease (stage IV: 44%). In this cohort, we identified a number of mutations and CNVs in several genes. Among these, oncogenic *PIK3CA* showed a high incidence of mutations in SDC patients (24.3%). The PI3K axis plays a critical role in tumorigenesis and the availability of small molecular inhibitors of this pathway renders it an attractive therapeutic target [13]. Recently, somatic mutations in *PIK3CA* have been identified in a variety of human tumors. Most of the reported mutations of *PIK3CA* are clustered in exons 9 and 20 of the gene [14,15], where three hotspot mutations (E542K, E545K, and H1047R) reside. Two of these hotspot mutations (E542K and E545K) are located in the helical domain (exon 9) whereas H1047R is located in the kinase domain (exon 20) of the PIK3CA protein. All three hotspot mutations have been proven to be oncogenic and are associated with poor clinical outcomes [16-18]. In our study, five patients had hotspot mutations in PI3KCA (one each with E542K and E545K, and three with H1047R). These results are consistent with recent studies that reported *PIK3CA* mutations in SDC [19-21]. In addition to gain-of-function mutations, *PIK3CA* is activated through gene amplifications in several cancers [18,22,23]. However, the nCounter CNV assay revealed no significant amplification of *PIK3CA* in SDC patients in this study (Figure 2). In addition to *PIK3CA* alterations, the phosphatase and tensin homolog (PTEN) also activates the PI3K pathway, and *PTEN* deletion was recently reported in SDC by FISH analysis [21,24]. We found 10 *PTEN* mutations in 5 out of 37 cases of SDC. Because of the limited amount of available tissue we could not determine whether these alterations of *PIK3CA* or *PTEN* are associated with aberrant protein expression and subsequent activation of the PI3K pathway. However, a previous study reported that *PIK3CA* amplification appears to be exceedingly rare in SDC, and that *PIK3CA* mutation may be seen simultaneously with *PTEN* loss [21]. Further study is required to assess whether *PIK3CA* mutation could be the predictor of poor prognosis in SDC.

In addition to the histologic resemblance, SDC is similar to breast ductal carcinoma regarding overexpression of ERBB2 and EGFR in many cases [25-30]. *ERBB2* gene amplification and protein overexpression occurs in 20-30% of breast cancers and is reported to be a significant predictor of poor prognosis [31]. Similarly, the majority of cases of SDC appear to strongly overexpress ERBB2. Consistent with previous studies, the majority of our cohort exhibited strong (copy number ≥10) amplification of *ERBB2*. The high concordance between immunohistochemistry and FISH analyses suggests that gene amplification is the most important mechanism for ERBB2 overexpression in this tumor [25,28,32,33]. These findings all point to a potential role for trastuzumab, an anti-ERBB2 antibody, in the treatment of SDC. Several studies have reported clinical results of treatment with trastuzumab in combination with chemotherapy [9,30,34]. In these studies, selected SDC cases with *ERBB2* amplification successfully treated with trastuzumab. However, until now *ERBB2* mutations had not been identified in patients with SDC. It is therefore noteworthy that we found *ERBB2* mutations in 4 of 37 patients. All these mutations were in exons 17–19 (Table 2). Although the functional relevance of these mutations has not been identified, patients with *ERBB2* mutation had a shorter median OS time than those without (20.8 vs. 70.9 months). This finding suggests that in a subset of SDC patients, the presence of *ERBB2* mutation represents a more attractive potential therapeutic target than *ERBB2* amplification.

It has been reported that *EGFR* overexpression is a poor prognostic factor in head and neck squamous cell carcinoma [35,36]. Moreover, cetuximab, an anti-EGFR monoclonal antibody, and/or gefitinib, an ATP-competitive EGFR tyrosine kinase inhibitor (TKI), have been studied in various types of salivary gland carcinoma [4,37-39], but not in SDC. EGFR activation and downstream signaling leads to increased cell proliferation and survival. Activating *EGFR* mutations are well described in non-small cell lung cancer and have been reported in various cancers of the salivary glands [25,39,40]. *EGFR* mutations are usually localized within two hotspots in the kinase domains, specifically a deletion in exon 19 and an L858R point mutation in exon 21. In addition, rare mutations have been discovered elsewhere in the kinase domain, including insertions in exon 20 [29]. In this study, we observed 7 *EGFR* mutations in 4 of 37 SDC patients. One of these mutations (V765M) is in exon 20 and the others (A859T, A864V, E865K, E866K, A871T, and G874S) are in exon 21. Although those are not well-known hotspot mutations, their close proximity to L858R suggests that they may play a role in SDC progression.

## Conclusions

In conclusion, we identified mutations in several genes including *PIK3CA*, *ERBB2*, and *EGFR* and significant gene amplification of *ERBB2* in SDC. Given the small number of cases in this study, the significance of specific mutation types, mechanism of gene amplification, and their relationship to other abnormalities has to be elucidated in larger studies. In Kaplan-Meier analysis using *PIK3CA* and *ERBB2* genes, the presence of mutations showed a higher correlation with overall survival than copy number variation. Although the importance of these mutations in SDC is still unchallenged, our results suggest that further studies of these mutations may identify a new therapeutic candidate and aid development of an effective treatment strategy in SDC.

## Consent

Written informed consent was obtained from the patient for the publication of this report and any accompanying images.

## Abbreviations

SDC: Salivary duct carcinoma
CNV: Copy number variation
SGC: Salivary gland cancer
ERBB2: Epidermal growth factor receptor 2
FISH: Fluorescence in situ hybridization
NGS: Next-generation sequencing
FFPE: Formalin-fixed paraffin-embedded tissue
COSMIC: Catalogue of Somatic Mutations in Cancer
DFS: Disease-free survival
PTEN: Phosphatase and tensin homolog
TKI: Tyrosine kinase inhibitor
RT: Radiotherapy
CCRT: Concurrent chemo-radiotherapy
DP: Docetaxel/Cisplatin
FP: 5FU/Cisplatin
CAP: Cyclophosphamide/Doxorubicin/Cisplatin
RFS: Recurrence-free survival
OS: Overall survival
